# A Study to Evaluate the Fracture Toughness of Denture Base Resin Reinforced With Nanomaterials as an Innovative Denture Base Resin

**DOI:** 10.1155/ijod/3689927

**Published:** 2026-10-21

**Authors:** Apurva Padhye, Sowmya M. Kumar, Arun M. Isloor, Muttanna Venkatesh, Chethan Hegde, Poornima G. Hiremath

**Affiliations:** ^1^ Department of Prosthodontics and Crown and Bridge Nitte (Deemed to be University) AB Shetty Memorial Institute of Dental Sciences (ABSMIDS) Mangaluru 575018 Karnataka India nitte.edu.in; ^2^ Membrane and Separation Technology Laboratory Department of Chemistry National Institute of Technology Surathkal Mangaluru 575025 Karnataka India nitk.ac.in; ^3^ Department of Chemical Engineering Siddaganga Institute of Technology Tumakuru 572103 Karnataka India sit.ac.in

**Keywords:** CaCO_3_ nanoparticles, denture base resins, fracture toughness, PMMA (polymethylmethacrylate), TiO_2_ nanotubes

## Abstract

**Objective:**

The most widely used denture base material, polymethylmethacrylate (PMMA) resin, by itself exhibits poor fracture toughness. The present study aimed to assess the fracture toughness of this denture base resin incorporating calcium carbonate nanoparticles (CNPs) and titanium dioxide nanotubes (TNTs) in varying concentrations.

**Materials and Methods:**

Eighty‐four heat‐cured PMMA samples were prepared, divided into seven groups with 12 specimens each. TNTs and CNPs were incorporated into PMMA at concentrations of 0.1%, 0.2% and 0.3%, while unmodified PMMA served as the control. For both materials and composites, the characterisation was conducted through Fourier‐transform infrared spectroscopy (FTIR), X‐ray diffraction, zeta potential (ZP) analysis, particle size (PS) measurement, scanning electron microscopy and energy‐dispersive X‐ray spectroscopy to ensure proper synthesis, integration and fabrication. The fracture toughness of each specimen was evaluated using a universal testing machine through a single‐edge‐notched three‐point bending test. Statistical analysis involved ANOVA followed by post hoc Tukey tests to determine significant differences.

**Results:**

Highest fracture toughness values were noted in PMMA with the 0.3% CaCO_3_ NPs (CNPs) (4.0 ± 0.19) and 0.3% TNTs (3.9 ± 0.47) reinforced groups when compared to 0.1%, 0.2% and control groups. This illustrated that there is a statistically significant difference in fracture toughness between the four groups.

**Conclusion:**

In comparison to other studies, an interesting finding was that the fracture toughness values at 0.3% were similar to those obtained at 1%. It suggested that a higher concentration may not always be necessary for optimum fracture toughness. Also, excessive concentrations may lead to particle clustering, which could compromise mechanical integrity. The obtained results suggest that nanomaterial inclusion is a promising approach to enhance the strength of denture base resins, but further long‐term clinical studies are necessary to validate their effectiveness.

## 1. Introduction

Oral rehabilitation with removable prostheses necessitates the usage of materials that are aesthetically pleasing, functionally adaptable and remain durable [1]. The most commonly used material for removable denture prosthesis is polymethylmethacrylate (PMMA) [2]. PMMA is preferred for its low density, aesthetic appeal, ease of manipulation, cost‐effectiveness and tailorable physical and mechanical properties. However, certain limitations of this material, such as low fracture toughness, greater incidence of porosity and polymerisation shrinkage and poor fatigue resistance, affect the overall quality of the prosthesis [3]. These limitations, when compounded with additional factors such as masticatory load and varying thickness of the denture, further contribute to the possibility of fractures of the teeth or along the midline of the removable denture prosthesis [4].

The development of newer materials such as polyamides, thermoplastic resins and three‐dimensional (3D)‐printed PMMA bases to overcome the limitations of conventional PMMA has additional limitations that hinder their effectiveness in fracture resistance [2]. Polyamide’s modulus is not sufficiently high to equal that of standard PMMA materials, requiring further modification to consistently produce better properties than current PMMA [5]. UV‐polymerised resins demonstrate higher plaque adhesion than PMMA with poorer surface quality on the tissue‐contacting side [6]. 3D‐printed denture bases show the most significant limitations, demonstrating the lowest mechanical performance [7]. This led to further exploration of alternative strategies, such as incorporating nanofiller (NF) particles such as titanium oxide (TiO_2_) and zirconia in conventional PMMA [8].

While the optimal filler levels of 2% to 6% have demonstrated improvement in flexural strength, fracture toughness and hardness, these high concentrations raise concerns on NPs agglomeration, aesthetic deterioration, handling difficulties and increased water absorption. At sub‐percentage levels, NP fillers disperse more evenly within the PMMA matrix, enhancing stress transfer and interfacial bonding without negatively affecting the clarity or handling properties essential for denture base materials [9]. Titanium dioxide nanotubes (TNTs) and calcium carbonate nanoparticles (CNPs) represent two underexplored alternatives with distinct potential advantages in this aspect. TNTs have demonstrated significant positive correlations with fracture toughness, flexural strength and hardness of denture base PMMA, yet existing investigations testing this at various concentrations of 1.5% to 5% report conflicting results [10].

Another material that has garnered interest is tungsten disulphide (WS_2_) NP research. While this material exhibited promising reinforcement properties at low concentrations (0.05%–0.1%), its scope was limited to flexural and impact testing without addressing fracture toughness—the most clinically relevant failure mode of PMMA dentures [11]. The existing literature has examined various mechanical properties of NP‐incorporated PMMA; however, direct evidence on fracture toughness remains limited and inconsistent across filler types and concentrations [12]. Furthermore, the evidence regarding newer alternative fillers such as TNTs and calcium carbonate NPs in lower concentrations is limited, precluding the ability to determine if meaningful reinforcement is achievable without the agglomeration, aesthetic compromise and handling difficulties associated with higher filler loadings. Therefore, the present study aimed to evaluate the efficacy of TNTs and calcium carbonate NPs at concentrations of 0.1%, 0.2% and 0.3% by weight on the fracture toughness levels of heat‐cured PMMA denture base resin. The null hypothesis stated that the incorporation of these fillers at the tested concentrations would not significantly alter fracture toughness relative to unmodified PMMA.

## 2. Materials and Methods

### 2.1. Materials

Titanium tetraisopropoxide [Ti (OC_3_H_7_)_4_] and acetic acid (CH_3_COOH) were procured from Sisco Research Laboratories Private Limited. Isopropyl alcohol (C_3_H_8_O) was supplied by MSB Chemicals Limited. Nitric acid (HNO_3_) was purchased from Pallav’s Chemicals and Solvents Private Limited. Calcium chloride (CaCl_2_) and Sodium carbonate (Na_2_CO_3_) were brought from Merck Life Sciences Pvt Ltd. PMMA was procured from heat‐curing acrylic Heracryl.

### 2.2. Methods

This in vitro study was conducted on PMMA denture base samples incorporated with nanotubes and NPs made according to ISO13586:2018 specifications. The institutional ethics committee granted ethical clearance for the study, and the Checklist for Reporting In‐vitro Studies (CRIS) guidelines [13] were followed for the reporting of this study. A prior literature review was used to determine the sample size. The samples were prepared according to the manufacturer’s guidelines, earlier research and ISO standards. The NPs and nanotubes were synthesised at the Membrane and Separation Technology Laboratory, Department of Chemistry, National Institute of Technology, India.

#### 2.2.1. Synthesis of TNTs

The sol–gel synthesis process was employed to prepare the TNTs. About 10 mL of isopropyl alcohol was mixed with 12 mL of titanium tetraisopropoxide. The resulting TiO_2_ solution (Sol) was further mixed with 150 mL of water and 5 mL of acetic acid. This mixture was heated to 80°C for 3 h with continuous stirring to prevent the aggregation of the sol particles. About 1 mL of concentrated HNO_3_ was additionally introduced to control the pH of the solution. The mixture was then dried in a vacuum oven for 10 h at 100°C [14], as detailed in Figure 1. TiO_2_ sol was formed by dissolving titanium tetraisopropoxide in isopropyl alcohol and then adding water and acetic acid. To adjust the pH, the mixture was heated at 80°C for 3 h with constant stirring and then concentrated HNO_3_ was added. The resultant sol was then dried in a vacuum oven at 100°C for 10 h to yield TNTs.

**Figure 1:**
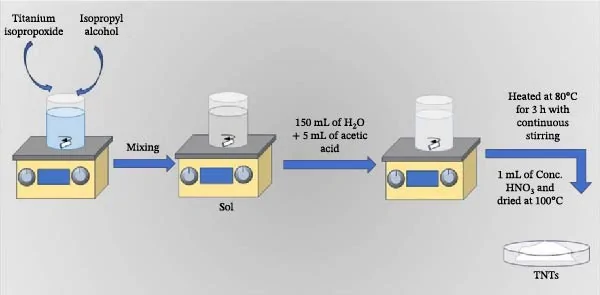
Schematic representation of the sol–gel synthesis process for preparing titanium dioxide nanotubes (TNTs), including mixing a titanium precursor with solvent, water, acetic acid, and nitric acid, followed by heating and drying.

#### 2.2.2. Synthesis of CNPs

0.01 M of analytical‐grade CaCl_2_ and Na_2_CO_3_ solutions were individually prepared using 50 mL of distilled water with continuous stirring and an aqueous Na_2_CO_3_ solution was added to the CaCl_2_ solution. A white precipitate was formed after 30 min of constant stirring at room temperature. The collected precipitate was filtered and repeatedly washed with distilled water. The resulting white CaCO_3_ NPs were first heated at 105°C for one hour in a hot air oven before being vacuum‐stored [14], as described in Figure 2.

**Figure 2:**
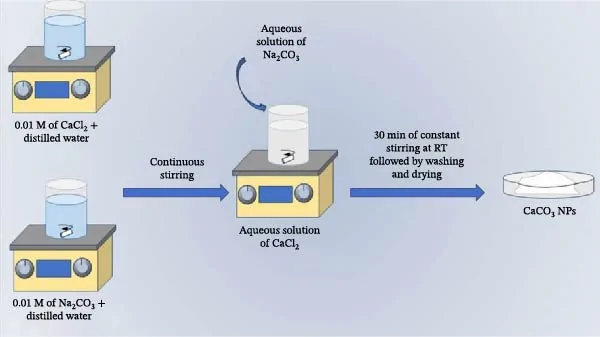
Schematic representation of the precipitation method used for the synthesis of calcium carbonate nanoparticles (CNPs), showing preparation of CaCl_2_ and Na_2_CO_3_ solutions, precipitation, filtration, washing, drying, and storage of the synthesised nanoparticles.

#### 2.2.3. Preparation of Study Samples

A total of 84 samples of heat‐cured PMMA blocks were prepared for this study. These were divided into seven groups containing 12 specimens each, wherein one group served as the control with no addition of NPs and the remaining six groups had NPs incorporated in varying concentrations of TNTs and CaCO_3_ NPs as follows (Figure 3):

**Figure 3:**
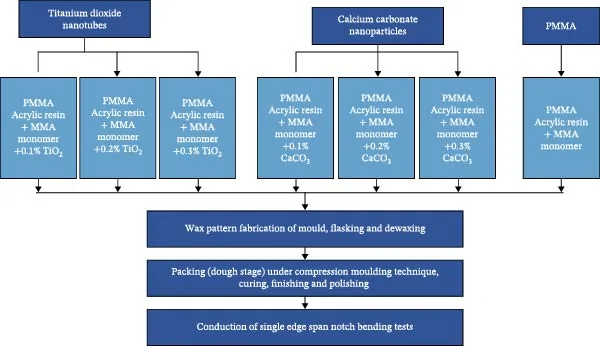
Schematic representation of the study design, showing the preparation of heat‐cured PMMA specimens and their allocation into seven experimental groups according to the type and concentration of incorporated nanofiller.

• Group I: PMMA + 0.1% wt. TNT.
• Group II: PMMA + 0.2% wt. TNT.
• Group III: PMMA + 0.3% wt. TNT
• Group IV: PMMA + 0.1% wt. CaCO_3._
• Group V: PMMA + 0.2% wt. CaCO_3._
• Group VI: PMMA + 0.3% wt. CaCO_3._
• Group VII: PMMA.

The fabrication of the samples was conducted in accordance with the ISO standards. Initially, wax patterns were fabricated using a split brass mould measuring 44 × 6 × 4 mm^3^, as shown in Figure 4. The wax patterns were flasked in type II dental stone, followed by dewaxing. Specific weight percentages of 0.1%, 0.2% and 0.3% were accurately measured for each sample and ground using a mechanical stirrer, followed by mortar–pestle grinding. This process aimed to break apart any clumps, decrease particle aggregation and produce a finely milled, uniform powder. The ground NFs were gradually mixed into the PMMA powder to ensure an even dispersion. The resulting mixture was then combined with the monomer liquid (methyl methacrylate [MMA]), placed into moulds and polymerised under carefully controlled thermal curing conditions. All the above‐mentioned samples were prepared at a weight of 10 g and mixed with 4.3 mL of MMA and flasked using the compression moulding technique. Finally, the sample was finished and polished, as shown in Figure 5. The fracture toughness of the sample is shown in Figure 6.

**Figure 4:**
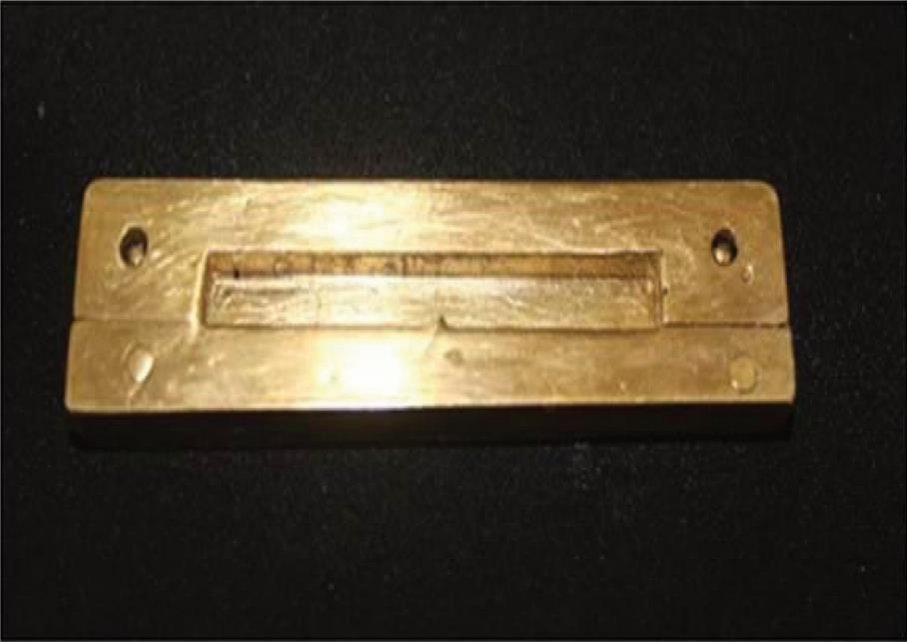
Brass split mould used for the fabrication of the PMMA specimens. The mould dimensions were 44 mm × 6 mm × 4 mm.

**Figure 5:**
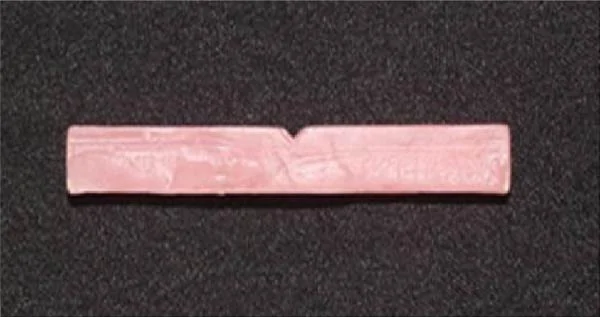
Finished and polished PMMA specimen, prepared for fracture toughness testing.

**Figure 6:**
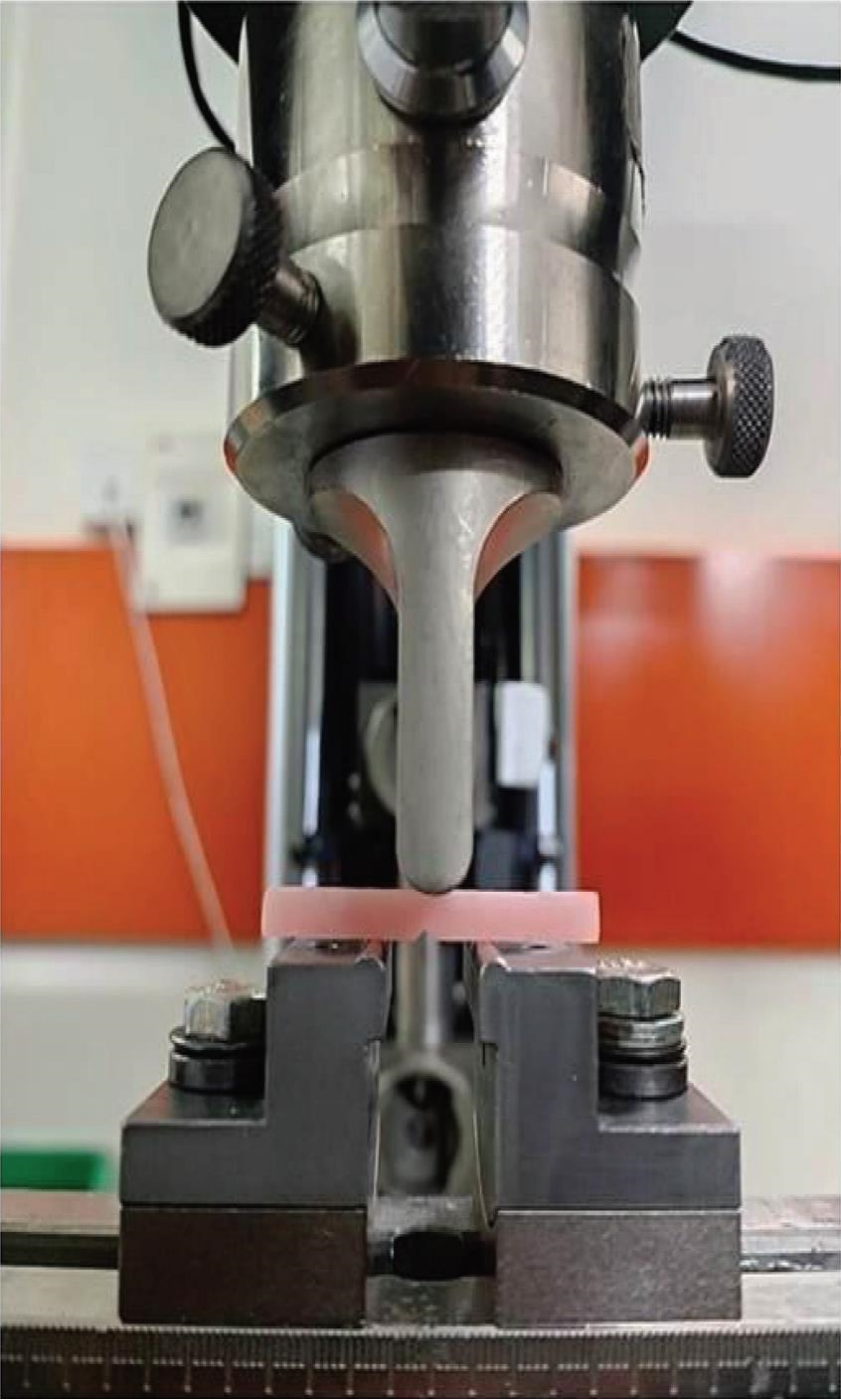
Universal testing equipment is used to measure the fracture toughness (K_IC_) of specimens with single‐edge notches. The maximum load at failure was noted for the K_IC_ computation after the specimen was loaded at a crosshead displacement rate of 1 mm/min till fracture. (Representative setup/procedure for fracture toughness analysis of the PMMA specimens using a universal testing machine).

Figure 3. Study design illustrating the experimental workflow. PMMA acrylic resin specimens were prepared by incorporating titanium dioxide (TiO_2_) nanotubes and calcium carbonate (CaCO_3_) NPs at concentrations of 0.1%, 0.2% and 0.3%, with unmodified PMMA serving as the control. Following material preparation, the specimens were processed and fabricated. The prepared specimens were subsequently subjected to single‐edge notched bending testing for the evaluation of fracture toughness and material characterisation.

#### 2.2.4. Measurement of Fracture Toughness

A universal testing machine with a 20 kN load cell and a crosshead speed of 1 mm/min of the specimen was used to test the fracture toughness of a single‐edge‐notched sample. A 1.2 mm sharp V‐shaped notch was incised in the 4 mm high sample 1. All samples were tested on the same day to reduce measurement bias. According to Equation (1), the plane strain fracture toughness (*K*_IC_) in MPa√m was determined using the greatest load (P) that caused a fracture [1, 2].

(1) Kic=3PL2BW32/ ×Y,

where ‘*P*’ is the peak load of fracture in Newton’s (N), ‘*L*’ is the span length (mm), ‘*B*’ is the specimen width (mm), *‘a’* represents notch depth and ‘*W*” is the specimen height (mm). The calibration function for the given geometry, ‘*Y*’, was calculated using Equation (2).

(2) Y=1.933.0714.5325.1125.80×aw12−×aw32+×aw52−× aw72+ ×aw92.

### 2.3. Material Characterisation

These descriptions clarify how individual fillers are successfully synthesised by detailing their functional groups, chemical bonds, crystallinity, phase purity, surface charge, particle size (PS) and distribution, morphology and elemental makeup. Specifically, the methods for creating TNTs and CNPs are outlined in Sections 2.2.1 and 2.2.2, which include information on precursor materials, reaction conditions and purification procedures. The process of integrating these NFs into the PMMA matrix is explained with exact concentrations, mixing protocols and curing parameters. Various characterisation techniques, such as Fourier‐transform infrared spectroscopy (FTIR), powder X‐ray diffraction (PXRD), zeta potential (ZP) analysis, PS measurement, scanning electron microscopy and energy‐dispersive spectroscopy, are described along with the respective instrument models, operational settings and analytical procedures to ensure that the results can be reliably reproduced.

#### 2.3.1. FTIR

This analysis was used to determine the functional groups and chemical bonding in the PMMA and the incorporated NFs (FTIR—Bruker Alpha) [14].

#### 2.3.2. PXRD

This study verifies the crystalline phases and structural integrity of the NPs within the composite (PXRD, Rigaku Corporation [Japan], Smart Lab) [14].

#### 2.3.3. ZP

This study helps analyse the surface charge properties and colloidal stability of the additives and composite dispersions (ZP, ZetaSizer Nano series, Malvern PANalytical) [15].

#### 2.3.4. PS Analyser

This analysis was performed to determine the hydrodynamic diameter (HD) and average PS distribution and to evaluate agglomeration tendencies (ZetaSizer Nano series) [15].

#### 2.3.5. Scanning Electron Microscope (SEM)–Energy Dispersive Spectroscopy (EDS)

This analysis helps to understand the surface morphology and elemental composition of the materials, confirming that the filler was evenly distributed (SEM, JEOL JCM 6000 Plus) [16, 17].

## 3. Statistical Analysis and Normality Test

The data obtained were entered into a Microsoft Excel spreadsheet and were subjected to statistical analysis. All analyses were conducted using the Statistical Package for the Social Sciences (Version 24, IBM, Chicago, USA). The Shapiro–Wilk test was used to assess the normality of quantitative variables. Since the data were normally distributed, parametric tests of significance were used. One‐way ANOVA was used to test whether there were statistically significant differences between the means of fracture toughness among seven independent groups. Continuous variables were expressed as mean ± standard deviation (SD). The level of significance was set at 5%. The assumption of normality was tested using the Shapiro–Wilk test.

One‐way ANOVA; Group I: PMMA acrylic resin + MMA monomer + 0.1% Ti0_2_; Group II: PMMA acrylic resin + MMA monomer + 0.2% Ti0_2_; Group III: PMMA acrylic resin + MMA monomer + 0.3% Ti0_2_; Group IV: PMMA acrylic resin + MMA monomer + 0.1% CaCO_3_; Group V: PMMA acrylic resin + MMA monomer + 0.2% CaCO_3_; Group VI: PMMA acrylic resin + MMA monomer + 0.3% CaCO_3_; Group VII: PMMA acrylic resin + MMA monomer.

Values are presented as mean ± SD. The effect size is reported as eta squared (*η* ^2^). The degrees of freedom for the ANOVA were 6 (between groups) and 77 (within groups).  ^∗∗^ *p* ≤ 0.001 is considered statistically significant.

## 4. Results

### 4.1. FTIR

The FTIR spectrum of CNPs revealed characteristic absorption bands at around 3430, 1590, 1368 and 668 cm^−1^. The CNPs@PMMA composite exhibited characteristic bands at ~3400, 2100,1590 and 1200 cm^−1^, along with the carbonate‐related band at 638 cm^−1^. The FTIR spectrum of the TNTs showed absorption bands at about 3430, 1590, 1385 and 652 cm^−1^. Following the incorporation of TNTs into the PMMA matrix, characteristic bands were noticed at around 2108 and 1400 cm^−1^, while the Ti‐O‐Ti vibration remained evident at ~652 cm^−1^. The broad absorption band at ~3430 cm ^−1^ was also retained in the TNTs@PMMA composite. The FTIR spectra of CNPs, CNPs@PMMA, TNTs and TNTs@PMMA are presented in Figure 7.

**Figure 7:**
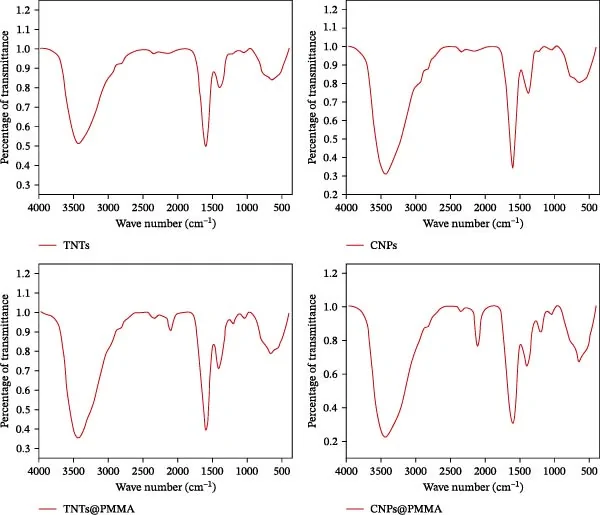
FTIR spectra of calcium carbonate nanoparticles, CNPs‐incorporated PMMA, titanium dioxide nanotubes, and TNTs‐incorporated PMMA showing the characteristic absorption bands of the fillers and their corresponding PMMA composites.

### 4.2. PXRD

The PXRD pattern of TNTs showed characteristic diffraction peaks at 20 values of 25.3°, 37.8°, 48.0°, 54.0° and 55.1°. No distinct peak corresponding to the rutile phase at ~27.4° was observed. The PXRD pattern of CNPs showed sharp and well‐defined diffraction peaks at 20 values of 23.1°, 29.5°, 36.0°, 39.5°, 43.2°, 47.5° and 48.6°. The most intense diffraction peak was observed at 29.5°. The PXRD patterns of CNPs and TNTs are represented in Figure 8.

**Figure 8:**
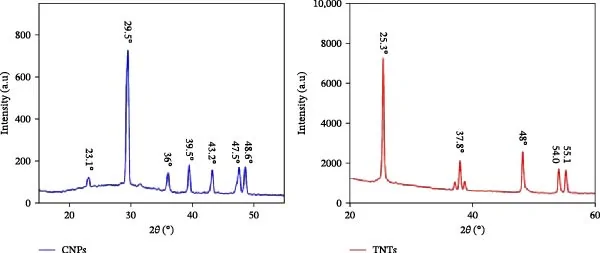
PXRD patterns of calcium carbonate nanoparticles and titanium dioxide nanotubes, showing the characteristic diffraction peaks associated with crystalline phases of the synthesised nanofillers.

### 4.3. ZP

The average ZP of CNPs was −22.1 mV, whereas that of the CNPs@PMMA composite was −18.6 mV. TNTs exhibited an average ZP of −34.1 mV, while the TNTs@PMMA composite showed a value of 32.6 mV, as recorded in the analysis. This is presented in Figure 9.

**Figure 9:**
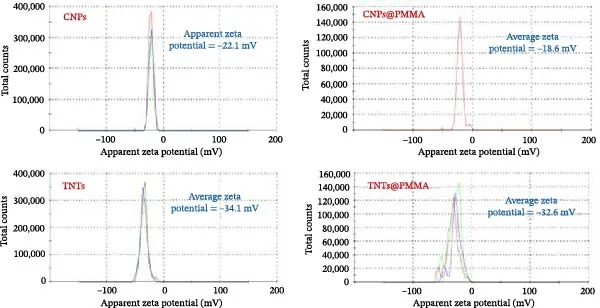
Zeta potential profiles of CNPs, CNPs@PMMA, TNTs and TNTs@PMMA, demonstrating the surface‐charge characteristics of the nanofillers before and after incorporation into the PMMA matrix.

### 4.4. PS

Dynamic light scattering analysis revealed an average PS of ~420.8 nm and a HD of around 1312 nm for CNPs. Following incorporation into PMMA, the average PS and HD were ~337.9 and 507.1 nm, respectively. For TNTs, the average PS was about 618 nm, with a corresponding HD of around 719.1 nm. After incorporation into PMMA, the average PS decreased to ~261.3 nm, whereas the HD increased to ~1123 nm. The PS distributions of CNPs, CNPs@PMMA, TNTs and TNTs@PMMA are presented in Figure 10.

**Figure 10:**
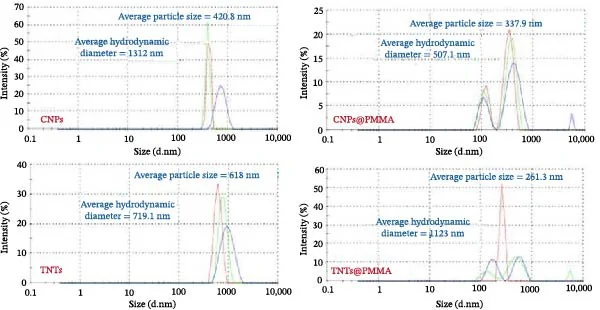
Particle‐size and hydrodynamic‐diameter distributions of CNPs, CNPs@PMMA, TNTs, and TNTs@PMMA determined by dynamic light scattering.

### 4.5. SEM–EDS

SEM examination of CNPs revealed a dense granular morphology, with particles showing a tendency to form clusters while retaining a nanoscale appearance. EDS analysis demonstrated carbon and oxygen contents of ~44.69% and 55.31%, respectively. The CNPs@PMMA specimens showed particles surrounded by the polymer matrix and a comparatively smoother surface morphology. The EDS spectra demonstrated carbon and oxygen signals. SEM examination of TNTs demonstrated a network of tubular structures. EDS analysis showed a predominant titanium signal with oxygen and minimal carbon signals. Following incorporation into PMMA, TNTs retained their tubular morphology, while the composite surfaces exhibited comparatively smoother features. EDS analysis demonstrated the presence of titanium, carbon and oxygen. SEM images and the corresponding EDS spectra are shown in Figure 11.

**Figure 11:**
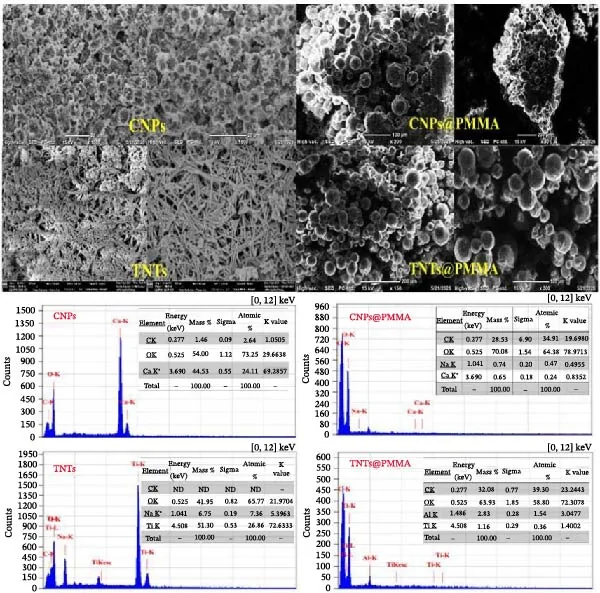
Representative SEM micrographs and corresponding EDS spectra of CNPs, CNPs@PMMA, TNTs and TNTs@PMMA, showing the morphology and elemental composition of the nanofillers and their PMMA composites.

### 4.6. Fracture Toughness of PMMA Nanocomposites (NCs) Reinforced With TNTs and CNPs at Low Concentrations

Descriptive statistics indicated variation in fracture toughness across the seven groups. Group VI had the highest mean fracture toughness (*M* = 4.00, SD = 0.20), followed by Group III (*M* = 3.92, SD = 0.47), Group II (*M* = 3.50, SD = 0.42), Group VII (*M* = 3.36, SD = 0.17), Group V (*M* = 3.05, SD = 0.20) and Group I (*M* = 2.97, SD = 0.22). Group IV exhibited the lowest mean fracture toughness (*M* = 2.88, SD = 0.75) (Figure 12).

**Figure 12:**
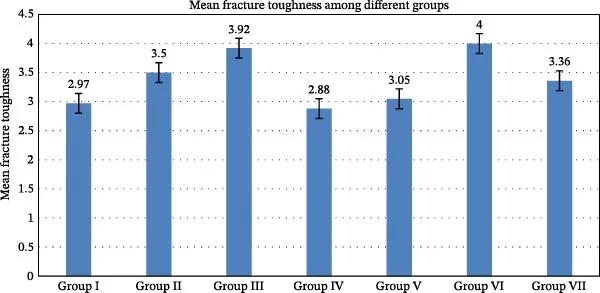
Mean fracture toughness values of the seven experimental groups. Group I: PMMA +0.1 wt.% TNT; Group II: PMMA +0.2 wt.% TNT; Group III: PMMA +0.3 wt.% TNT; Group IV: PMMA +0.1 wt.% CNP; Group V: PMMA +0.2 wt% CNP; Group VI: PMMA +0.3 wt.% CNP; Group VII: unmodified PMMA. Values are presented as mean fracture toughness for each group.

The analysis revealed a statistically significant difference among groups, *F* (6,77) = 15.26, *p* ≤ 0.001. The effect size was large, with eta squared (*η* ^2^) = 0.54, indicating that 54% of the variance in fracture toughness was due to differences between groups. Group VI exhibited the highest mean fracture toughness, whereas Group IV showed the lowest. These findings suggest substantial intergroup differences in fracture toughness (Table 1).

**Table 1:** Intergroup comparison for fracture toughness (*n* = 12 per group).

Groups	Mean	SD	*η*2	*F*	*p* ≤ value
Group I	2.97	0.22	0.54	15.26	0.001
Group II	3.50	0.42			
Group III	3.92	0.47			
Group IV	2.88	0.75			
Group V	3.05	0.20			
Group VI	4.00	0.20			
Group VII	3.36	0.17			

Reported values include mean differences with corresponding 95% confidence intervals (CI) and exact *p*‐values.  ^∗∗^ *p* ≤ 0.001,  ^∗^ *p*  < 0.05 is considered statistically significant. Tukey’s post hoc analysis revealed multiple statistically significant pairwise differences in fracture toughness among the seven groups. Group I demonstrated significantly lower fracture toughness compared to Group II (mean difference  =   − 0.53, *p* = .026), Group III (mean difference  =   − 0.95, *p* ≤ .001) and Group VI (mean difference  =   − 1.03, *p* ≤ .001). No significant differences were observed between Group I and Groups IV, V or VII.

Group II exhibited significantly higher toughness than Group IV (mean difference  =  0.61, *p* = .004), but significantly lower toughness than Group VI (mean difference  =   − 0.50, *p* = .044). Other comparisons involving Group II were non‐significant.

Group III consistently outperformed several groups, showing higher toughness compared to Group I (mean difference = 0.95, *p* ≤ .001),   Group IV (mean difference = 1.04, *p* ≤ .001), Group V (mean difference = 0.87, *p* ≤ .001) and Group VII (mean difference = 0.56, *p* = .016). Differences between Group III and Group II or Group VI were not statistically significant.

Group IV displayed significantly lower fracture toughness compared to Group II (mean difference = −0.61, *p* = 0.004), Group III (mean difference = −1.04, *p* ≤ 0.001) and Group VI (mean difference = −1.12, *p* ≤ 0.001). The remaining comparisons for Group IV were non‐significant.

Group V was significantly weaker than Group III (mean difference = −0.87, *p* ≤ 0.001) and Group VI (mean difference = −0.95, *p* ≤ 0.001), but did not differ significantly from Groups I, II, IV, or VII.

Group VI consistently demonstrated the highest fracture toughness values, significantly exceeding Groups I (mean difference = 1.03, *p* ≤ 0.001), II (mean difference = 0.50, *p* = 0.044), IV (mean difference = 1.12, *p* ≤ 0.001), V (mean difference = 0.95, *p* ≤ 0.001) and VII (mean difference = 0.64, *p* = 0.003). The difference between Group VI and Group III was not significant.

Finally, Group VII exhibited significantly lower fracture toughness compared to Group III (mean difference = −0.56, *p* = 0.016) and Group VI (mean difference = −0.64, *p* = 0.003). Other comparisons involving Group VII were non‐significant. Hence, results indicated that the data were normally distributed, *W* (84) = 0.98, *p* = 32. The non‐significant *p*‐value (*p* > 0.05) indicates that we accept the null hypothesis.

## 5. Discussion

The FTIR spectrum of CNPs displayed notable absorption features characteristic of calcite. A broad peak around 3430 cm^−1^ indicated O‐H stretching vibrations associated with adsorbed water molecules. The peak near ~1368 cm^−1^ is linked to C‐H or C‐O bending vibrations, while the band at approximately ~1590 cm^−1^ corresponds to asymmetric *C* = C stretching vibrations and also overlaps with the carboxylate species. The peak at ~668 represented the C‐H out‐of‐plane bending of the C‐H group. In the case of the CNPs@PMMA composite, the FTIR spectrum shows characteristic PMMA peaks, including a prominent band at 3400 cm^−1^ attributed to O‐H stretching, 2100 cm^−1^ is due to alkyne/nitrile or gas phase background, 1590 cm^−1^ is attributed to *C* = C stretching vibration and another at 1200 cm^−1^ assigned to C‐O‐C stretching. Additionally, carbonate bands observed at 638 cm^−1^ confirm the out‐of‐plane bending mode. The spectrum of TNTs features a broad absorption band at 3430 cm^−1^ due to O‐H stretching vibrations on surface hydroxyl groups. The bands at 1590 cm^−1^ correspond to the physically adsorbed H‐O‐H bending vibration. The peak at 1385 cm^−1^ is the characteristic peak of the deformation vibration of Ti‐OH. The strong peak at 652 cm^−1^ indicates Ti‐O‐Ti lattice vibrations, confirming the anatase crystalline phase of TiO_2_ nanotubes. When TNTs are incorporated into the PMMA matrix, the FTIR spectrum retains key features, with bands at 2108 cm^−1^ associated with *C* = O and ~ 1400 cm^−1^ attributed to the aliphatic backbone. The Ti‐O‐Ti lattice vibration at about 652 cm^−1^ is clearly visible, confirming TNT integration. The broad O‐H stretch at ~3430 cm^−1^ persists, indicating hydroxyl groups on TNT surfaces. A slight shift in the *C* = O band compared to pure PMMA suggests hydrogen bonding or dipolar interactions between PMMA ester groups and TNT surface hydroxyls, which are expected to enhance mechanical properties. This incorporation potentially promotes better compatibility and dispersion; these characteristic peaks are consistent with previously reported literature studies [16, 18], as illustrated in Figure 7.

Analysis of the PXRD data revealed that the TNTs displayed clear characteristic peaks associated with the anatase phase of TiO_2_. The most prominent peak appeared at a 2*θ* angle of 25.3°, with additional notable peaks at 37.8°, 48.0°, 54.0° and 55.1°. The lack of rutile‐specific peaks, particularly around 27.4°, indicates that the calcination at 600°C successfully stabilised the anatase structure, which is preferred due to its higher photocatalytic efficiency and biocompatibility compared to rutile. The diffraction pattern closely matches the reported references, confirming the material’s purity and crystalline quality. Likewise, the PXRD pattern of the calcium CNPs displayed sharp, well‐defined peaks, indicative of a crystalline phase. Key peaks appeared at 23.1°, 29.5°, 36.0°, 39.5°, 43.2°, 47.5° and 48.6°. The strongest peak at 29.5° corresponds to the calcite form of CaCO_3_, with no signs of the aragonite phase. The pattern is consistent with previously established references [19–21], supporting the phase purity of the samples, as illustrated in Figure 8.

The ZP of CNPs was approximately −22.1 mV, indicating a moderately negative surface charge. This negative charge primarily results from carbonate groups and adsorbed ions on the NP surface. Such a value indicates good colloidal stability as electrostatic repulsion minimises particle aggregation. When these CNPs are embedded in a PMMA matrix, their average ZP slightly decreases to −18.6 mV, likely due to the partial shielding of the carbonate groups by the polymer chains. Although less negative than the pure CNPs, the composite still displays sufficient electrostatic repulsion, complemented by steric stabilisation from the PMMA matrix, to maintain dispersion stability. The TNTs show a more negative mean ZP of −34.1 mV, which is consistent with the presence of hydroxyl groups and oxygen vacancies on the TiO_2_ surface. This highly negative charge contributes to electrostatic stabilisation, reducing the tendency for particle aggregation in suspension and promoting uniform dispersion within polymer matrices. After incorporation into PMMA, their average ZP slightly drops to −32.6 mV, a change attributed to polymer adsorption on the NT surface that partially masks the hydroxyl groups. Despite this decrease, the TNTs remain highly negatively charged, ensuring good colloidal stability. The combined effects of electrostatic and steric stabilisation mechanisms facilitate the uniform distribution of TNTs within the PMMA matrix, as illustrated in Figure 9.

The dynamic light scattering analysis revealed that CNPs have an average PS of ~420.8 nm, with a HD of around 1312 nm. The much larger HD compared to the PS indicates the presence of hydration layers and potential aggregation in suspension, a common phenomenon for CNPs due to their surface charge and ionic interactions. When these CNPs are embedded in PMMA, their average PS decreases to about 337.9 nm, and the HD reduces to 507.1 nm, suggesting that the PMMA chains effectively coat the NPs, providing stabilisation and reducing agglomeration. The polymer matrix’s steric hindrance enhances dispersion and results in a narrower PS distribution compared to pure CNPs. TNTs show an average PS of 618 nm with a HD of ~719.1 nm. The similarity in these values indicates that TNTs are less susceptible to extensive aggregation than CNPs, likely due to their higher negative surface charge, as confirmed by ZP measurements. Their morphology also contributes to a broader size distribution yet maintains colloidal stability. Incorporating TNTs into PMMA reduces their average PS further to about 261.3 nm, while the HD increases to around 1123 nm. This decrease in core size suggests effective interaction and stabilisation with the polymer, while the larger HD reflects the formation of polymer‐tube complexes. Overall, these interactions imply strong interfacial bonding between TNTs and PMMA, which is anticipated to improve the mechanical properties of the composite, as illustrated in Figure 10.

SEM images of the CNPs revealed a dense, granular structure typical of carbonate NMs. Although the particles tended to aggregate, they preserved their nanoscale dimensions. EDS confirmed the presence of carbon and oxygen, with ~44.69% carbon and 55.31% oxygen by mass, consistent with the expected composition of CNPs. No extraneous spectral peaks were detected, indicating a high purity level of the synthesised particles. When incorporated into a PMMA matrix, the NPs appeared as encapsulated particles within the polymer and their surface exhibited a smoother texture compared to pure particles, suggesting effective coating. The EDS spectra showed peaks for both carbon and oxygen, with a slight increase in the carbon content attributed to the PMMA matrix.

The results show that CNPs were evenly distributed within the polymer matrix, indicating good compatibility. The TNTs formed a network of tubular structures, confirming successful synthesis through calcination. Their uniform, elongated morphology is typical of TNTs. EDS analysis revealed a dominant titanium signal with minimal carbon or oxygen contamination, suggesting phase purity. When incorporated into the PMMA matrix, TNTs maintained their structural integrity and surface imaging showed smoother surfaces and better dispersion, likely due to the polymer coating. EDS spectra confirmed the presence of titanium, carbon and oxygen, verifying PMMA incorporation and the high titanium content indicated TNTs remained within the composite. Additional carbon and oxygen signals demonstrated effective polymer integration, as shown in Figure 11. Homogeneous filler dispersion was confirmed through combined SEM and EDS analysis. SEM images at multiple magnifications visually verified the even distribution of CNPs and TNTs within the PMMA matrix. EDS analysis of selected regions confirmed a uniform distribution of calcium, titanium, carbon and oxygen, with no clustering.

ZP measurements and PS analysis showed stable negative ZP values, indicating electrostatic stabilisation, along with narrow PS distributions in the composite compared to pure fillers—both signs of effective dispersion and reduced aggregation. These findings strongly support the uniform distribution of fillers in the PMMA matrix, essential for the consistent and reliable assessment of mechanical properties. The PSs for CaCO3 NPs (around 420.8 nm) and their composites (~337.9 nm) and TNTs (~618 nm) and their composites (~261.3 nm) were selected for this study based on their improved efficacy and minimal adverse effects. Future studies evaluating minimal NF concentrations in heat‐cured PMMA denture bases and their clinical relevance could guide in vivo research. The use of TNTs and spherical NPs demonstrated that larger surface areas enhance polymer bonding and reduce crack propagation. However, limitations include the lack of comparative assessment of curing cycles, which may affect the fracture toughness results. Additionally, all tests were performed under dry conditions without thermocycling or water ageing, limiting clinical applicability since intraoral water absorption can plasticise the PMMA matrix and weaken its mechanical properties over time.

Overall, Group VI exhibited the highest fracture toughness among all groups. Group VI values were significantly greater than those of Groups I, II, IV, V and VII (*p* < 0.05), confirming its superior performance. Group III also demonstrated relatively high toughness, significantly exceeding Groups IV, V and VII but did not statistically differ from Group VI (Table 2). The selected concentrations were guided by previous studies on polymer NCs, which consistently show that low levels of additive incorporation can enhance both mechanical and thermal performance without causing particle agglomeration or hindering the processability. Research involving carbon CNPs and TNTs in acrylic and polymer matrices indicates that concentrations between 0.1 and 0.3 wt% facilitate strong filler‐matrix interfacial bonding, improve stress transfer and reduce clustering. Such effects tend to become more significant at higher loadings, typically around 0.5–1 wt%.

**Table 2:** Tukey’s post hoc pairwise comparison of fracture toughness among the seven groups.

Reference group	Comparison group	Mean difference	95% CI		*p ≤* value
			Lower limit	Upper limit	
Group I	Group II	−0.53 ^∗^	−1.02	−0.03	0.026
	Group III	−0.95 ^∗∗^	−1.45	−0.45	0.001
	Group VI	−1.03 ^∗∗^	−1.53	−0.54	0.001
Group II	Group IV	0.61 ^∗^	0.12	1.11	0.004
	Group VI	−0.50 ^∗^	−1.00	−0.008	0.044
Group III	Group IV	1.04 ^∗∗^	0.54	1.53	0.001
	Group V	0.87 ^∗∗^	0.37	1.36	0.001
	Group VII	0.56 ^∗^	0.06	1.05	0.016
Group IV	Group VI	−1.12 ^∗∗^	−1.61	−0.62	0.001
Group V	Group VI	−0.95 ^∗∗^	−1.45	−0.45	0.001
Group VI	Group VII	0.64 ^∗^	0.14	1.14	0.003

^∗^Indicates a statistically difference at *p* ≤ 0.05.

^∗∗^Indicates a statistically significant difference at *p* ≤ 0.001.

Masticatory forces exert significant pressure, and the inadequate strength of removable dentures could result in crack propagation, predisposing dentures to fractures, especially along the midline of maxillary dentures. Fracture toughness, expressed as the plane strain stress intensity factor KIc, is the mechanical parameter most directly associated with this failure mode and represents a clinically relevant benchmark for denture base material performance [22, 23]. An ideal denture base material should resist the impact of functional load while safeguarding residual ridges. Although numerous attempts to explore NPs to enhance the mechanical properties of the denture base material have been made, the findings remain inconsistent. Therefore, this study aimed to address the effectiveness of low‐concentration NPs (TNT and Calcium carbonate NPs) at 0.1%, 0.2% and 0.3% by weight on the fracture toughness of heat‐cured PMMA resin. Both TNTs and CaCO_3_ NPs were chosen for their potential reinforcing properties and their light colour, which would help minimise the adverse effects on the aesthetics of PMMA denture bases [24, 25]. The current study results were compared with previously reported literature studies as detailed in (Table 3). Since the inclusion of NPs had a significant impact on the fracture toughness levels of heat‐cure PMMA resin, the null hypothesis was rejected.

**Table 3:** Comparative fracture toughness values of NPs‐reinforced PMMA denture base resins.

Nanofillers	Additives concentrations (%)	Reported fracture toughness (MPa√m)	Ref.
CaCO_3_ NPs/TiO_2_ NTs	**0.1–0.3**	**0.3% CaCO**_**3**_ **NPs: 4 ± 0.19;** **0.3% TiO_2_ TNs: 3.9 ± 0.47**	**Current study**
TiO_2_ NTs	2.5–5	5% TNTs > control (*p* = 0.002)	[16]
ZrO2NPs/TiO_2_ NPs	1.5–7	3% ZrO_2_ exhibited the highest fracture toughness; E‐glass fibre improved the impact strength	[22]
WS_2_ NPs	0.05 −0.1	0.1% flexural strength *p* = 0.106 (No fracture toughness reported)	[11]
TiO_2_ NPs in PMMA and PEEK	2–3	(3% TiO_2_) PMAA 1.62 ± 064; (3% TiO_2_) PEEK 4.91 ± 0.30	[26]

*Note:* Bold values represent the findings of the present study.

The highest level of fracture toughness was obtained with 0.3% wt of both TNTs and CaCO_3_ NPs, implying that a minimum of 0.3% wt of NP fillers may be required to improve the fracture toughness of the heat‐cure PMMA resin denture base. These findings were similar to the study conducted by Alhareb et al. [27], who evaluated the impact strength and fracture toughness of zirconium and TiO_2_‐reinforced PMMA denture bases and concluded that at a higher concentration of 5%–7%, a decrease in fracture toughness values was observed. This decrease could be explained by the degree of filler agglomeration and the potential for voids to form stress concentration points within the polymer matrix [26]. These factors disrupt the uniformity of the stress distribution and, as a result, reduce the degree of interfacial bonding between the matrix and NPs.

## 6. Conclusion

The study identified a clear relationship between the fracture toughness and reinforcement of PMMA. Specifically, groups reinforced with 0.3% TiO_2_ NTs and CaCO_3_ NPs showed a significant increase in toughness compared to the unmodified control, with CaCO_3_ exceeding 4 MPa√m. These findings indicate that a 0.3% concentration effectively enhances mechanical properties. Nonetheless, additional research, including long‐term in vivo studies, biocompatibility tests and clinical trials, is necessary to confirm the safety and effectiveness for routine dental or biomedical applications. Further investigations may explore how NP reinforcement influences the mechanical strength and crack resistance of PMMA denture bases.

**Nomenclature**
TNTs@PMMA: Titanium dioxidenanotubes@polymethylmethacrylate
CNPs@PMMA: Calcium carbonatenanoparticles@polymethylmethacrylate
PMMA: Polymethylmethacrylate
CNPs: Calcium carbonate nanoparticles
TNTs: Titanium dioxide nanotubes
ANOVA: Analysis of variance
NFs: Nanofillers
NTs: Nanotubes
K_IC_: Plane strain fracture toughness/critical stress intensity factor
FTIR: Fourier‐transform infrared spectroscopy
PXRD: Powder X‐ray diffraction
ZP: Zeta potential
PS: Particle size
SEM: Scanning electron microscope
EDS: Energy dispersive spectroscopy
NPs: Nanoparticles
HD: hydrodynamic diameter
NRs: Nanorods
NMs: Nanomaterials.

## Funding

No funding was received for this manuscript.

## Conflicts of Interest

The authors declare no conflicts of interest.

## Data Availability

The data that support the findings of this study are available upon request from the corresponding author. The data are not publicly available due to privacy or ethical restrictions.
